# Multi-omic integration identifies broad drug resistance mechanisms and strategies to therapeutically reprogram cancer cells

**DOI:** 10.1016/j.isci.2025.114293

**Published:** 2025-11-28

**Authors:** Ian Mersich, Brian S.J. Blagg, Aktar Ali

**Affiliations:** 1 Department of Chemistry and Biochemistry, University of Notre Dame, Notre Dame, IN 46556, USA; 2 Warren Family Research Center for Drug Discovery and Development, University of Notre Dame, Notre Dame, IN 46556, USA; 3 Harper Cancer Research Institute, University of Notre Dame, Notre Dame, IN 46556, USA

**Keywords:** Health sciences

## Abstract

Broad drug resistance arises from diverse transcriptional, metabolic, and genetic adaptations, yet the unifying features that sustain cross-resistant phenotypes remain unclear. We developed an integrative framework combining PRISM drug-response data with transcriptomic, metabolomic, and mutational profiles to define the molecular programs associated with broad resistance and to nominate compounds capable of reversing them. Resistant cell lines exhibited coordinated activation of extracellular matrix remodeling, stress-adaptation pathways, and survival signaling, with NFE2L2 emerging as a central regulatory hub linking upstream mutations to oxidative-stress transcriptional programs. Multi-omic analyses revealed metabolic reprogramming as a conserved feature of resistance, and patient cohort analyses showed that resistance-associated alterations correlated with shorter progression-free survival. Computational perturbagen screening identified compounds predicted to counteract these transcriptional signatures, converging on regulators of NFE2L2 activity. Experimental testing confirmed that rosiglitazone reduced NFE2L2-associated gene expression and re-sensitized resistant cells to chemotherapy, demonstrating a scalable strategy for rational phenotypic reprogramming.

## Highlights

• Multi-omic integration with drug screening data defines mechanisms of drug resistance
• Network modeling identifies NFE2L2 as a key regulator of resistance programs
• Altered resistance programs in patients have been shown to be negatively correlated with survival
• Perturbagen screening identifies compounds that reprogram resistance programs

## Graphical abstract

**Figure:**
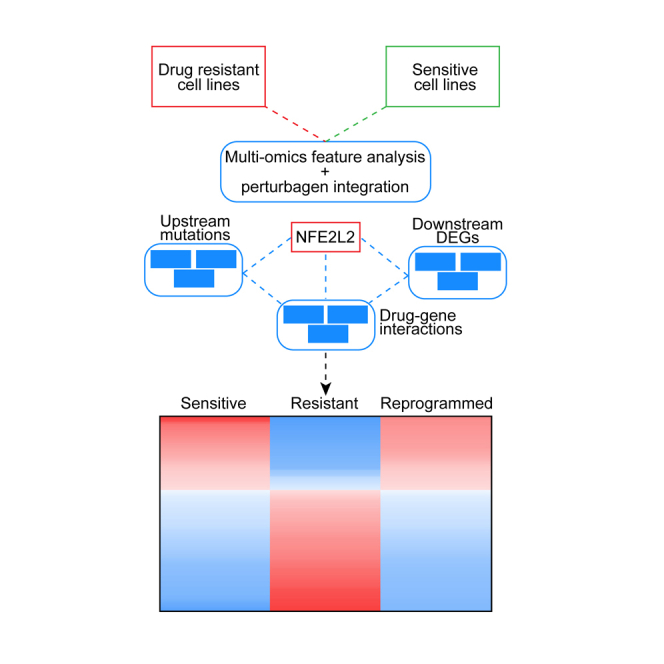

## Introduction

Drug resistance remains a central barrier to effective cancer treatment, arising through diverse and often multifactorial mechanisms that allow cancer cells to evade the cytotoxic effects of therapy.^1^^,^^2^^,^^3^ Mechanisms driving resistance include enhanced drug efflux via transporters,^4^^,^^5^^,^^6^^,^^7^ alterations in drug targets,^8^ dysregulation of apoptosis pathways,^9^^,^^10^^,^^11^ and activation of stress-response signaling networks.^12^^,^^13^^,^^14^ Additionally, cancer stem-like cells, cellular quiescence, epithelial-to-mesenchymal transition, and heterogeneous cell populations have been implicated in mediating broad and persistent resistance phenotypes acro cancer types.^15^^,^^16^^,^^17^^,^^18^^,^^19^ Understanding and overcoming these resistance mechanisms is essential for advancing durable cancer therapies and improving patient outcomes.^20^^,^^21^

The continued emergence of new mechanisms of multidrug resistance, including adaptations in metabolic pathways and microenvironmental interactions, further underscores the complexity of drug resistance in cancer and the need for integrative strategies to address it.^22^ Advances in large-scale functional genomics and pharmacologic screening efforts, such as the Cancer Dependency Map^23^ and PRISM Repurposing datasets,^24^ have enabled systematic mapping of drug responses across thousands of genetically characterized cancer cell lines, providing a framework to investigate the molecular correlates of drug sensitivity and resistance at unprecedented scale.^25^^,^^26^^,^^27^^,^^28^^,^^29^ These resources facilitate integrated approaches that combine drug response profiling with multi-omic data, including transcriptomics, mutational landscapes, and metabolomics, to identify pathways and gene networks that mediate resistance phenotypes.^30^^,^^31^^,^^32^^,^^33^ The availability of comprehensive clinical and genomic resources, such as the Catalog of Somatic Mutations in Cancer (COSMIC),^34^ The Cancer Genome Atlas (TCGA),^35^ and ClinVar,^36^ alongside bioinformatics toolkits for drug resistance analysis,^37^ further expands the capacity to systematically interrogate the genetic basis of therapeutic failure and prioritize candidate vulnerabilities for intervention.

Despite these advances, overcoming broad and multi-class drug resistance across mechanistically diverse therapies remains a formidable challenge in oncology. Efforts to systematically map resistance mechanisms and identify re-sensitization strategies are critical for the development of rational drug combinations and targeted interventions that can restore sensitivity in resistant cancers.^24^^,^^38^^,^^39^^,^^40^^,^^41^^,^^42^^,^^43^^,^^44^^,^^45^^,^^46^ Here, we leverage large-scale drug sensitivity datasets in conjunction with transcriptomic, mutational, and metabolomic data to characterize the molecular features of broad drug resistance in cancer cell lines and to identify candidate compounds predicted to reverse resistant phenotypes through integrated perturbagen screening. By combining computational network analyses with pharmacogenomic data, this study aims to advance rational strategies for overcoming drug resistance and guiding the development of targeted therapies in resistant cancer models.

## Results

### Systematic identification of broadly sensitive and resistant cancer cell lines

**Figure 1:**
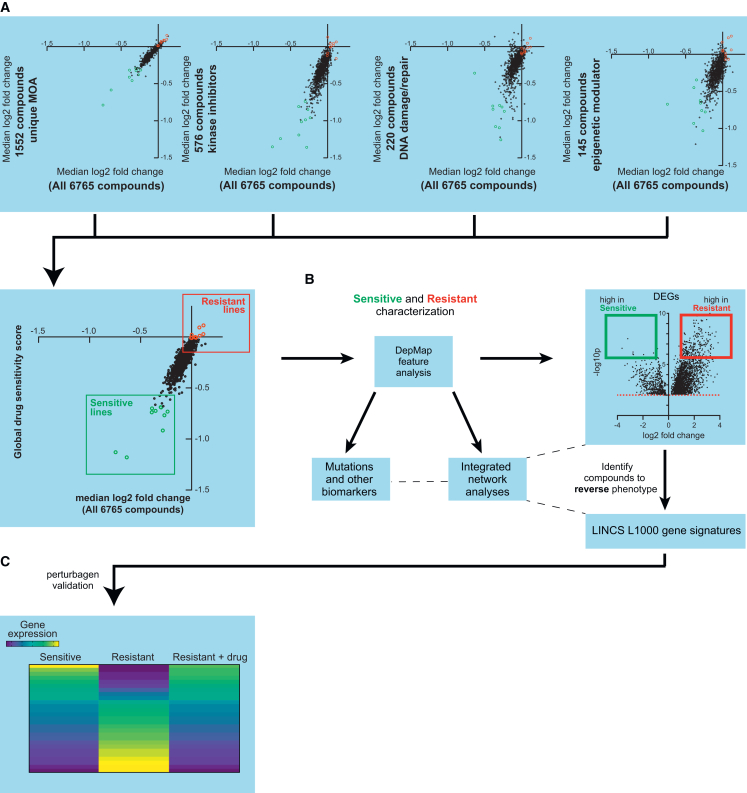
Systematic identification of broadly sensitive and resistant cancer cell lines (A) Overview of drug-sensitivity scoring using PRISM repurposing data. For each cell line, median log_2_ fold-change (LFC) values were computed across mechanistically distinct drug classes, and class-level medians were averaged to generate a composite drug-sensitivity score. This composite approach was benchmarked for variance reduction and stability across classes (see Figure S1). (B) Workflow for classifying cell lines as sensitive or resistant and integrating genomic and transcriptomic profiles to characterize these populations. (C) Strategy for leveraging resistance-associated DEGs and perturbagen-screening data to identify compounds predicted to reverse the drug-resistant phenotype.

To explore shared mechanisms of broad-spectrum drug resistance, we developed a computational framework to classify cancer cell lines based on their global drug sensitivity profiles. The experimental workflow is outlined in Figures 1A–1C. Using PRISM Repurposing data from the DepMap portal we computed a drug sensitivity score for each cell line by calculating the median log2 fold change for all compounds using subsets of mechanistically distinct drug classes (e.g., compounds with unique mechanism of action, kinase inhibitors, DNA-damaging agents, and epigenetic modulators), and averaged the class-level medians to generate a single composite sensitivity score (Figure 1A; Table S1). These scores were visualized against the overall drug response distribution across 6765 compounds (median log2 fold change for all compounds), revealing a broad range of sensitivities across cell lines. To ensure that the composite sensitivity score reflected a balanced representation of drug responses rather than being dominated by specific classes, we compared it to a simple median log_2_ fold-change (LFC) computed across all compounds. Because drug classes in PRISM vary widely in compound number (e.g., kinase inhibitors are overrepresented relative to epigenetic modulators), a global median LFC disproportionately reflects the most common mechanisms of action. In contrast, our class-weighted composite approach normalizes across mechanistic diversity by first calculating class-specific medians and then averaging them, thereby assigning equal weight to each drug class. We used a rank-based approach to quantify variance in drug sensitivity across mechanistic classes, which confirmed that this method yields stable rankings across classes (Figure S1), supporting its use as a generalizable measure of broad-spectrum drug sensitivity.

Our workflow also utilizes transcriptomic (RNA-seq) and mutation profiles from DepMap with flexible significance and magnitude thresholds to identify resistance-associated features and enriched gene sets. Integrated analyses linking mutations to differentially expressed genes (DEGs), were interrogated using network-level approaches (Figure 1B), and in parallel, DEGs from resistant lines were queried against the LINCS L1000 database to identify perturbagens predicted to reverse resistance-associated expression signatures (Figure 1C).

### Transcriptomic analysis reveals gene signatures and pathways associated with broad resistance

**Figure 2:**
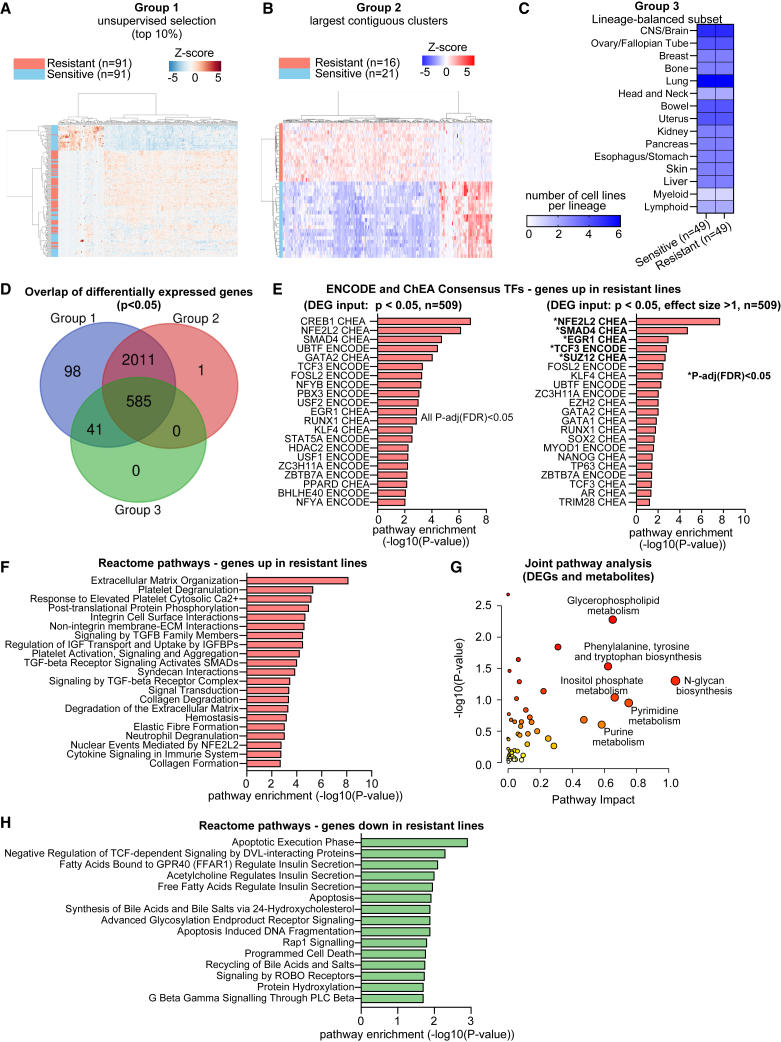
Transcriptomic analysis reveals gene signatures and pathways associated with broad resistance (A–C) Schematic of three strategies for defining resistant and sensitive groups: Group 1, top and bottom 10% by drug sensitivity score (*n* = 91 sensitive and *n* = 91 resistant models); Group 2, largest contiguous transcriptional clusters from Group 1 (*n* = 21 sensitive and *n* = 16 resistant models); Group 3, lineage-balanced subsets (*n* = 49 sensitive and *n* = 49 resistant models). (D) Overlap of differentially expressed genes (DEGs) identified across the three independent resistant-versus-sensitive comparison strategies. 585 DEGs (*p* < 0.05) were shared across all three groups. All 585 DEGs had adjusted *p*-value (FDR) < 0.05 in Group 1, 563 of 585 in Group 2, and none in the lineage-balanced Group 3. (E) Transcription factor enrichment analysis (Enrichr) of DEGs upregulated in resistant lines. Left panel: DEGs filtered by *p* < 0.05. All listed transcription factors were significant after Benjamini-Hochberg correction (FDR <0.05). Right panel: DEGs filtered by *p* < 0.05 and effect size >1, all listed transcription factors were significant (*p* < 0.05) and ∗ indicated for transcription factors that were significant after FDR correction. (F) Reactome pathway enrichment analysis (Enrichr) of genes upregulated in resistant lines. (G) Joint pathway analysis (MetaboAnalyst) integrating DEGs (*n* = 585) and differentially abundant metabolites (*n* = 30). Each point represents a pathway. The x axis indicates pathway impact (derived from topology analysis), the y axis shows the -log_10_*p* value from enrichment analysis, point size is proportional to pathway impact, and point color corresponds to statistical significance (darker red = more significant). -log_10_*p* value >1.3 ∼ *p* < 0.05. (H) Reactome pathway enrichment analysis (Enrichr) of genes downregulated in resistant lines.

To investigate transcriptional programs underlying broad drug resistance, we performed differential expression analyses between resistant and sensitive cell lines using three complementary comparison strategies (Figures 2A–2C; Table S2). Group 1 was defined by selecting the top and bottom 10% of lines based on composite drug-sensitivity scores (*n* = 91 in each group; Figure 2A). Group 2 consisted of transcriptionally clustered lines from Group 1 that tightly segregated with their respective phenotypes, thereby minimizing intra-group heterogeneity (Figure 2B). Group 3 was constructed to control for lineage-specific biases by including only cancer types represented in both resistant and sensitive categories.

Although there was some lineage imbalance in Groups 1 and 2 (Figure S2A), univariate regression analyses did not reveal any statistically significant association between drug-sensitivity score and lineage (Figure S2B). Additional metadata variables such as collection site, disease subtype, and media conditions were also evaluated (Figures S2C–S2F; Tables S3 and S4). Several metadata features showed significant associations with the composite sensitivity score after FDR correction (23/507, q < 0.05; Table S4). Most significant features were related to the “Oncotree primary disease” classifier, whereas broader contextual variables such as lineage, subtype, and collection site were not. Because disease-type features often correlate with lineage, we additionally performed a lineage-balanced analysis (Group 3), which yielded 585 overlapping DEGs (*p* < 0.05) across all grouping strategies (Figure 2D), confirming that key resistance-associated signatures are not driven by lineage composition alone. All three grouping strategies showed robust stratification between sensitive and resistant populations (Figures S2G–S2I).

DEGs were identified for each resistant-versus-sensitive comparison group, and correlation analyses were conducted between gene expression and drug-sensitivity scores (Table S5). A set of 585 DEGs (*p* < 0.05) was shared across all three comparison strategies, representing the most robust gene-expression markers for broadly resistant cell lines (Figure 2D; Table S6). Among these, all genes met FDR <0.05 in Group 1, 563 of 585 in Group 2, and none in the lineage-balanced Group 3, though all remained nominally significant (*p* < 0.05).

Next, we performed functional enrichment analyses with flexible gene-set inputs for exploratory purposes. Transcription-factor enrichment analysis (Enrichr) of genes upregulated in resistant lines identified multiple upstream regulators previously linked to drug resistance (Figure 2E; Table S7). The left panel shows enrichment results using DEGs with a *p* < 0.05 cutoff, where all listed transcription factors remained significant after FDR correction (q < 0.05). The right panel applies a more stringent filter for input DEGs (*p* < 0.05 and effect size >1), yielding a reduced set of regulators that retained biological coherence. Notably, NFE2L2 (NRF2), a central regulator of oxidative-stress and multidrug-resistance programs, was consistently among the top-ranked factors under both filtering strategies. We retained the more relaxed exploratory strategy for DEG inputs (*p* < 0.05) for additional downstream analyses. Reactome enrichment of these genes (Figure 2F; Table S8) identified several biologically relevant pathways associated with resistance, including extracellular matrix (ECM) organization, platelet activation, TGF-beta receptor signaling, IGF transport regulation, neutrophil degranulation, and Nuclear Events Mediated by NFE2L2. These results highlight a coordinated transcriptional program involving matrix remodeling, stress signaling, and survival mechanisms.

To complement the transcriptomic findings, we compared the metabolomics profiles between sensitive and resistant lines. Metabolites showing differential abundance were combined with DEGs in a joint pathway analysis using MetaboAnalyst (Figure 2G; Tables S9 and S10). Several pathways emerged as significantly enriched, including N-glycan biosynthesis, amino acid biosynthesis (e.g., phenylalanine, tyrosine, valine, leucine), purine and pyrimidine metabolism, glycosaminoglycan degradation, and inositol phosphate metabolism. These results suggest that broad drug resistance is accompanied by widespread metabolic reprogramming affecting both nucleotide and amino acid metabolism.

Finally, Reactome pathway analysis of genes downregulated in resistant lines (Figure 2H) revealed enrichment for apoptosis-related programs, indicating that the suppression of cell death machinery is a key feature of resistance. Taken together, these results suggest that broadly resistant cell lines undergo coordinated remodeling of stress adaptation, metabolic reprogramming, and enhancement of survival programs.

### Mutation signatures and regulatory network analyses associated with broad drug resistance

**Figure 3:**
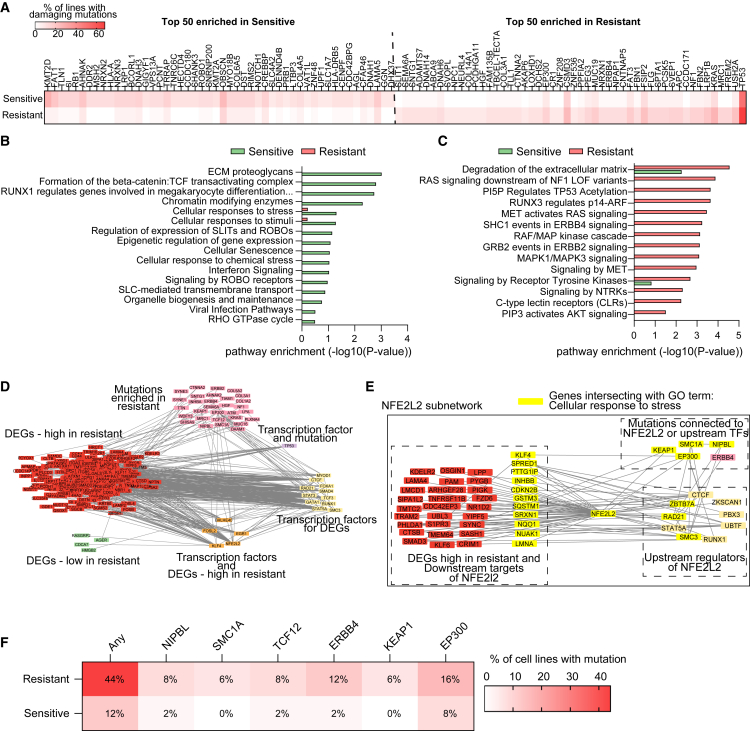
Mutation signatures and regulatory network analyses associated with broad drug resistance (A) Gene-level mutation frequency differences between the 50 most resistant and 50 most sensitive cell lines. Top 50 genes ranked by group skew are shown. (B and C) Pathway enrichment analyses of top genes preferentially mutated in sensitive (B) and resistant (C) lines. (D) Integrative gene-regulator network combining DEGs, enriched transcription factors, and recurrently mutated genes. Edges represent protein-protein interactions for genes (nodes) from STRING (high confidence, direct interactions) and transcription factor – target interactions (from transcription factor enrichment analysis). (E) NFE2L2-centered subnetwork highlightes upstream regulators and downstream targets linked to stress response and matrix remodeling. (F) Heatmap shows the percentage of resistant and sensitive lines with mutations in NFE2L2-linked genes, illustrating convergent and non-overlapping mutation patterns contributing to resistance.

To investigate mutation patterns associated with broad drug resistance, we applied two complementary analytical strategies. In the first approach, we compared gene-level mutation frequency differences between the 50 most resistant and 50 most sensitive cell lines (Figure 3A). For each gene, we calculated a “group skew score” as the difference in the percentage of mutated resistant lines versus sensitive lines. The top-ranked genes from each group were independently subjected to pathway enrichment analyses (Figures 3B and 3C).

Genes more frequently mutated in sensitive lines were enriched in pathways related to chromatin remodeling, cell cycle regulation, and cellular stress response, including chromatin modifying enzymes, RUNX1-regulated transcription, epigenetic regulation of gene expression, and cellular senescence, along with signaling cascades such as Rho GTPase signaling, ROBO receptor signaling, and interferon signaling (Figure 3B; Table S11). Interestingly, this gene set also implicated pathways tied to SARS-CoV-2 infection and viral host interactions, suggesting that heightened innate immune signaling or cellular stress priming may sensitize these lines to therapy.

Conversely, genes more commonly mutated in resistant lines were enriched for growth factor signaling, matrix remodeling, and canonical oncogenic pathways, including RAF/MAPK signaling cascade, PI3K/AKT signaling, RTK signaling (e.g., MET, ERBB4, and NTRK), and TP53 regulatory pathways (Figure 3C; Table S11). Notably, multiple pathways tied to extracellular matrix organization and immune system modulation were enriched, highlighting potential links between microenvironmental remodeling and broad drug resistance.

As a complementary strategy, we performed sample-specific pathway enrichment using the full mutation profiles of an individual cell line (Figure S3A). Each sample was scored for pathway mutation burden based on -log10 *p*-values, and hierarchical clustering revealed distinct separation between resistant and sensitive lines (Figure S3B). To quantify these differences, we calculated pathway-level skew scores, defined as the difference in the proportion of enriched resistant versus sensitive samples, adjusted for the mean enrichment significance across each group. A volcano plot (Figure S3C; Table S12) highlighted key pathways skewed toward either phenotype. Sensitive lines exhibited enrichment for pathways such as second messenger signaling, WNT/TCF-dependent transcription, histone demethylation, and cell death regulation. Resistant lines showed enrichment in RUNX3/p14-ARF signaling, PDGF/MET/FGFR signaling, collagen biosynthesis, and non-integrin ECM interactions. Notably, glycosaminoglycan degradation, a pathway involved in ECM turnover, was significantly enriched in resistant lines in both the mutation-based pathway analysis (Figure S3C) and the prior joint transcriptomic-metabolomic pathway analysis (Figure 2G; Table S10). Genes related to ECM proteoglycans were also enriched in the mutation analyses; however, the direction of enrichment differed depending on the analytical scale.

In the gene-level analysis, enrichment was based on the subset of genes showing the strongest mutation bias toward either sensitive or resistant populations (Figure 3B). This approach highlights which genes disproportionately harbor mutations in one group versus the other. In contrast, the pathway-level analysis incorporated all detected mutations across genes within each pathway for every cell line, capturing overall mutation burden rather than directionality. This per-sample aggregation identifies pathways recurrently affected in resistant phenotypes even when individual mutations are present in different populations with a group skew, or when distributed across multiple genes. The observed divergence between gene- and pathway-level enrichments therefore reflects differences in analytical granularity, whether enrichment emphasizes specific high-bias genes or aggregate pathway involvement. Together, these complementary analyses underscore the complex and multifaceted role of ECM remodeling in mediating broad drug-resistance phenotypes.

To further investigate the mechanistic connections between mutational patterns and resistance phenotypes, we constructed an integrative gene-regulator network (Figure 3D), incorporating frequently mutated genes, differentially expressed genes (DEGs), and transcription factors (TFs) enriched from ENCODE and ChEA databases. This network identified hubs of regulatory convergence, linking upstream mutations to downstream transcriptional changes. One prominent hub was NFE2L2, a key regulator of oxidative stress response and cell survival. A focused subnetwork centered on NFE2L2 (Figure 3E) revealed associations with xenobiotic metabolism, ECM remodeling, and antioxidant defenses. Several infrequently mutated genes (KEAP1, EP300, ERBB4, TCF12, SMC1A, and NIPBL) were functionally linked to this hub. Although each gene individually had a low mutation frequency of only 6–16%, 44% of resistant lines harbored at least one mutation in this set (Figure 3F). These mutations typically occurred in a mutually exclusive manner, supporting a model in which diverse genetic alterations converge to dysregulate NFE2L2 signaling, thereby promoting resistance across multiple drug classes.

### Clinical relevance of resistance-associated mutations in patient cohorts

**Figure 4:**
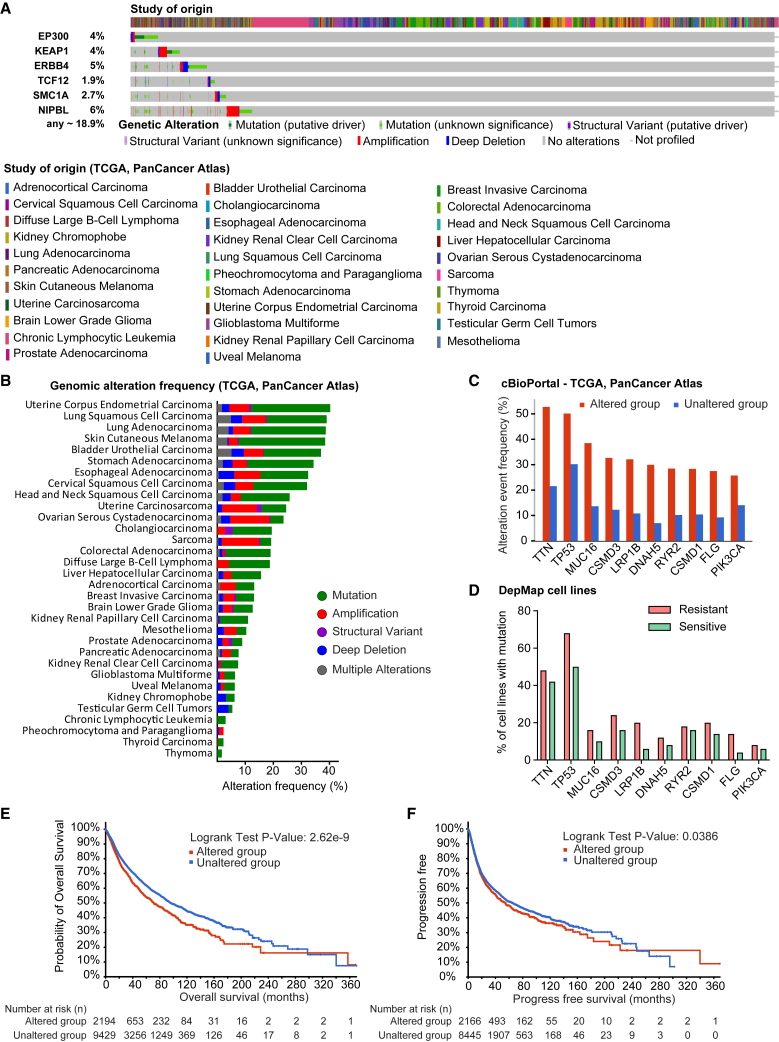
Clinical relevance of resistance-associated mutations in patient cohorts (A) Total number of TCGA PanCancer patient samples harbors genetic alterations in at least one of the six resistance-associated genes (KEAP1, EP300, ERBB4, TCF12, SMC1A, and NIPBL). (B) Frequency of patients with these mutations across individual cancer types. (C) The most frequently co-occurring genomic alterations in patients with resistance-associated mutations. (D) Comparison of co-occurring mutation frequencies between resistant and sensitive DepMap cell lines. (E and F) Kaplan-Meier survival analysis (log-rank test) shows reduced overall survival (E) and progression-free survival (F) in patients harboring resistance-associated mutations.

To assess the clinical relevance of mutations identified in resistant cell lines, we queried cBioPortal using data from over 11,000 patients across 32 TCGA PanCancer Atlas cohorts. Figure 4A shows the total number of patients with genomic alterations in at least one of the six genes identified in our mutation network analysis (KEAP1, EP300, ERBB4, TCF12, SMC1A, and NIPBL). These genes were selected based on their collective enrichment in resistant cell lines and their convergence on NFE2L2 and related cellular stress response pathways.

Patient samples were stratified into “altered” (with at least one genomic alteration in these genes) and “unaltered” groups, revealing broad distributions of alterations across diverse cancer types. Figure 4B presents the percentage of altered patients within each TCGA cohort, highlighting higher mutation frequencies in specific cancer types, including lung adenocarcinoma and uterine corpus endometrial carcinoma.

We next identified the most frequently co-occurring genomic alterations in the altered group (Figure 4C). Many of these co-altered genes are involved in cell signaling, transcriptional regulation, or chromatin remodeling. We then compared the mutation frequency of these co-occurring genes with our earlier analysis in sensitive and resistant lines from the DepMap dataset (Figure 4D). These alterations were more prevalent in resistant lines, further supporting their relevance to the resistance phenotype.

Finally, we analyzed clinical outcomes between altered and unaltered patient groups. Kaplan-Meier survival curves revealed significantly reduced overall survival (OS; Figure 4E) and progression-free survival (PFS; Figure 4F) in patients harboring mutations in the selected resistance-associated genes. While decreased OS likely reflects more aggressive tumor biology, the shortened PFS supports a role for these mutations in driving therapeutic resistance in human cancers.

### Drug perturbation analyses reveal re-sensitization strategies and mechanistic convergence on resistance pathways

**Figure 5:**
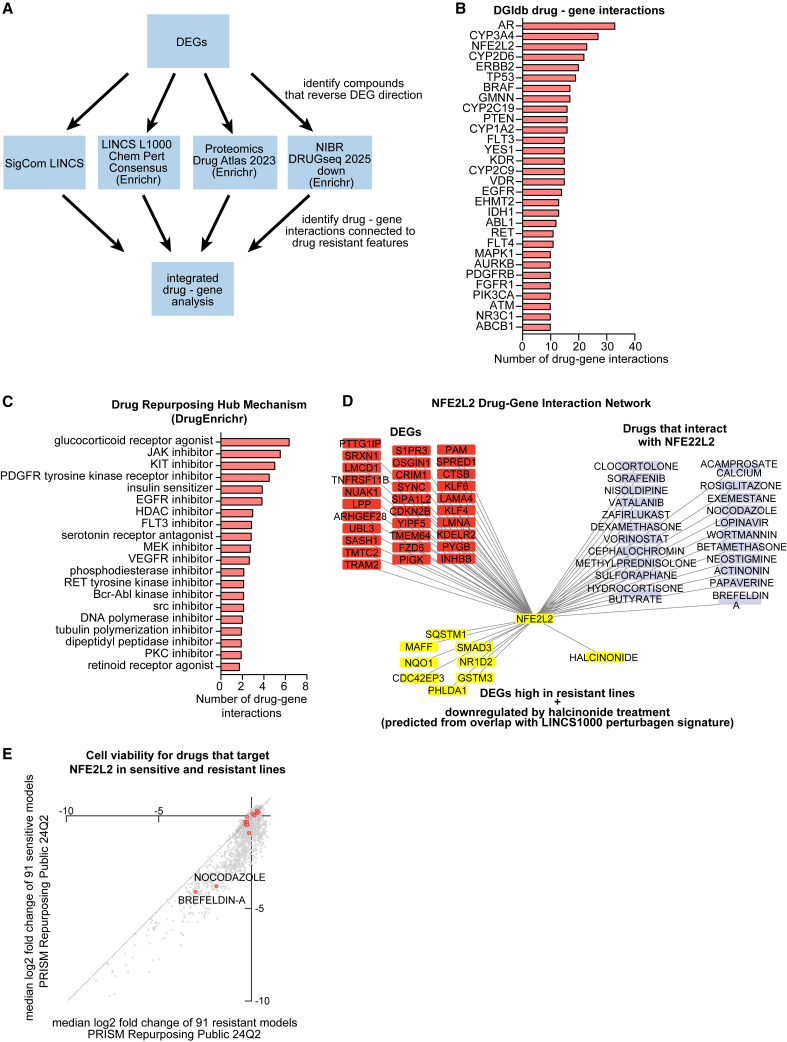
Drug perturbation analyses reveal re-sensitization strategies and mechanistic convergence on resistance pathways (A) Schematic outlines the perturbagen screening workflow using resistance-associated DEGs (*p* < 0.05) to identify compounds predicted to reverse resistance-associated gene expression signatures. Candidate compounds were selected from the top 100 hits across LINCS L1000, DRUGseq, and Drug Atlas libraries, all of which exhibited significant enrichment, adjusted *p* < 0.05 (BH-FDR). (B) Top drug-gene interactions identified across datasets from A (Top 100 from each library), highlighting top drug-gene interactions by number of compounds per gene. (C) DrugEnrichR analysis of compounds (from panels A and B) for mechanisms of action. Plot represents all mechanisms with significant enrichment *p* < 0.05 (BH-FDR). (D) Integrated drug-gene network mapping candidate compounds to resistance-associated genes and transcription factors. Cytoscape interactions (edges) based on DGIdb drug-gene (nodes) interactions, and PPIs between genes. NFE2L2-centered subnetwork highlighting NFE2L2 as a target of halcinonide and DEGs predicted to be downregulated by halcinonide treatment that are also NFE2L2 targets overexpressed in resistant lines. (E) DepMap PRISM analysis of candidate compound activity, using viability as a readout (log 2-fold change) across sensitive and resistant lines, to identify bioactive compounds.

To identify candidate compounds capable of reversing resistance phenotypes, we queried multiple drug perturbation gene set libraries using resistance-associated DEGs, prioritizing compounds predicted to repress overexpressed genes in resistant lines and restore those suppressed (Figure 5A; Table S13).

Top candidate compounds were mapped to their known gene targets using DGIdb. Several targets recurred across datasets, most notably NFE2L2, previously identified as a central transcriptional regulator in resistant lines. Other frequently targeted genes included ABCB1 (a canonical mediator of multidrug resistance through drug efflux), as well as AR, TP53, BRAF, EGFR, and various cytochrome P450 enzymes (Figure 5B). This convergence on stress response regulators (e.g., NFE2L2), drug metabolism genes (e.g., CYP3A4 and CYP2D6), and efflux transporters (e.g., ABCB1) underscores a coherent mechanistic axis underlying broad resistance that may be pharmacologically reversible.

Using DrugEnrichR, we performed pathway enrichment of the candidate compounds, which revealed shared mechanisms of action. The most enriched drug classes included glucocorticoid receptor agonists, JAK inhibitors, KIT and EGFR tyrosine kinase inhibitors, HDAC inhibitors, and MEK inhibitors (Figure 5C). These drug classes closely align with resistance-associated pathways identified in earlier analyses, such as stress response, chromatin remodeling, and RTK/MAPK signaling.

We then constructed a drug-gene interaction network in Cytoscape to visualize connections between resistance-targeting compounds and resistance-associated genes (Figure S4). This network integrated DEGs and mutations from resistant lines with drug-target relationships, highlighting central hubs such as NFE2L2, EGFR, and TP53, which had also emerged in earlier transcriptomic and mutational analyses (see Figures 3G and 3H).

To further refine therapeutic strategies, we developed an NFE2L2-centric subnetwork highlighting its transcriptional targets upregulated in resistant lines and the compounds predicted to modulate these genes (Figure 5D). Among these, halcinonide showed strong overlap with NFE2L2 transcriptional targets and demonstrated the ability to downregulate them, supporting its potential to disrupt NFE2L2-driven resistance programs.

Finally, we leveraged DepMap PRISM repurposing data to assess the functional impact of candidate compounds in sensitive and resistant cell lines. Median log2 fold-change values were plotted for each compound in sensitive versus resistant lines (Figure 5E). Brefeldin A and nocodazole exhibited the highest overall impact on cell viability in resistant lines, suggesting these compounds effectively engage targets within resistant cells and may have the capacity to shift transcriptional programs toward a more sensitive phenotype. While not selective for resistant cells, this pharmacologic activity indicates a functional readout in resistant populations, providing a rationale for further exploration of these compounds in re-sensitization studies.

### Experimental validation supports predicted perturbagen screening

**Figure 6:**
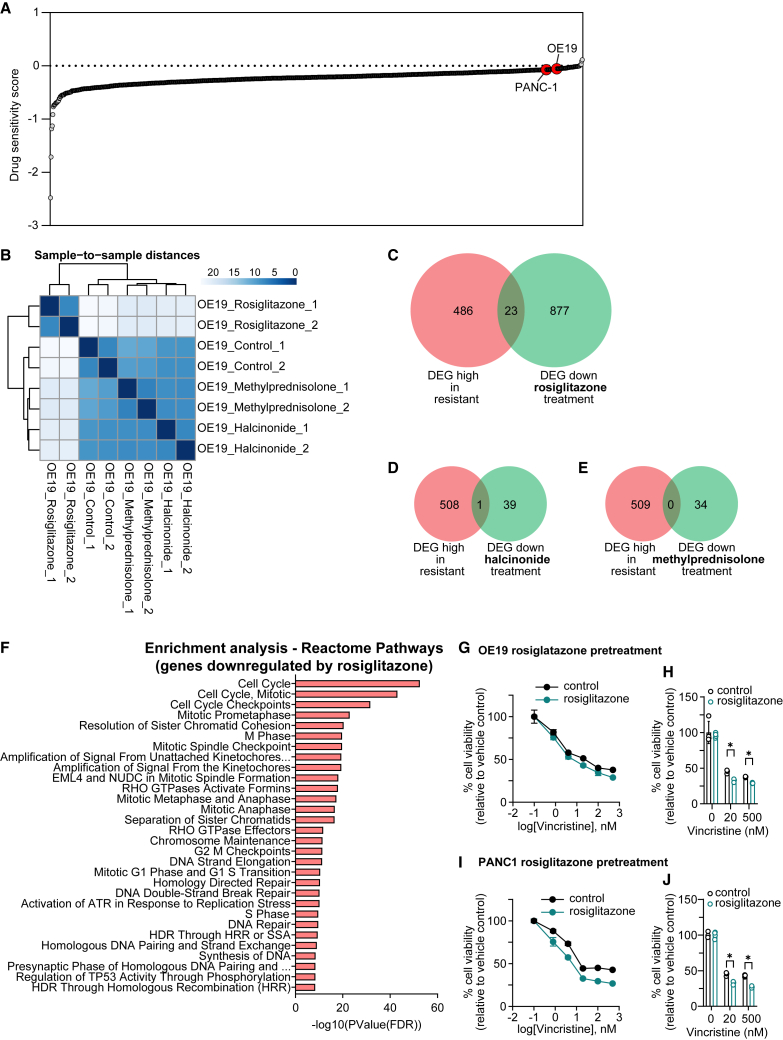
Experimental validation supports predicted perturbagen screening (A) Waterfall plot of drug sensitivity scores from DepMap cell lines. Two resistant lines available in our laboratory, OE19 and PANC-1, were selected for experimental validation. (B) Sample-to-sample distances. Heatmap generated with the DeSeq2 software packages shows the Euclidean distances between the samples. (C–E) Venn diagrams show overlap between genes upregulated in resistant lines and those downregulated following halcinonide, methylprednisolone, or rosiglitazone treatment. DEGs filtered by *p* < 0.05 and log_2_FC < −0.5. (F) Functional enrichment analysis of genes downregulated by rosiglitazone (*p* < 0.05 and log_2_FC < −0.5). (G–J) Dose-response curves (G and I) for OE19 and PANC-1 cells following 24-h pretreatment with rosiglitazone (1 μM) before 72 h vincristine exposure. (H and J) Bar graphs for two representative vincristine concentrations, data are represented as mean ± SD (∗*p* < 0.05; multiple unpaired t tests with FDR correction).

We next tested three compounds connected to NFE2L2 through curated DGIdb interactions and predicted to repress NFE2L2 transcriptional targets based on LINCS1000 perturbation signatures (Table S14). Compounds were selected from our available screening library and tested in cell lines previously identified as broadly drug-resistant (Figure 6A). To assess the predicted repression of NFE2L2-dependent transcriptional networks, OE19 esophageal adenocarcinoma cells were treated with halcinonide, methylprednisolone, or rosiglitazone (1 μM, 24 h) followed by RNA-seq profiling (*n* = 2 per condition). Unsupervised analyses revealed a clear treatment-induced divergence: treated samples separated distinctly from controls by principal component analysis (Figure S5A), and sample-to-sample distance clustering showed the most pronounced shift in cells treated with rosiglitazone (Figure 6B).

To determine whether perturbagen treatment reversed gene expression programs associated with broad drug resistance, we compared genes upregulated in resistant lines to those downregulated following treatment (Venn diagrams, Figures S5B–S5D). Using a stringent threshold (|log_2_FC| > 1), only a small number of overlapping genes were detected—one in halcinonide, none in methylprednisolone, and five in rosiglitazone. When applying a relaxed cutoff (|log_2_FC| > 0.5), overlap with rosiglitazone increased substantially (Figures 6C–6E). Fisher’s exact tests indicated a modest enrichment trend for rosiglitazone (odds ratio = 1.42, P = 0.07; Table S15), whereas the other compounds showed no significant overlap. These results suggest that rosiglitazone induces partial reversal of the transcriptional state associated with broad drug resistance.

Functional enrichment analysis of genes downregulated by rosiglitazone (Figure 6F) revealed convergence with several pathways upregulated in resistant lines (see Figure 2). Specifically, resistant cells displayed the activation of vesicle trafficking and Rho GTPase/ECM remodeling, while rosiglitazone promoted the reciprocal regulation of these systems through retrograde Golgi transport and Rho GTPase effector modulation. Additionally, pathways associated with TGF-β signaling and metabolic reprogramming in resistant cells corresponded to TP53-mediated checkpoint and DNA-repair programs following rosiglitazone treatment, indicating a shift from stress-tolerant to checkpoint-restorative cellular states. Together, these findings highlight a coordinated, inverse relationship between resistance-associated and rosiglitazone-responsive transcriptional networks.

To test whether transcriptional reprogramming translated to functional re-sensitization, we pre-treated OE19 cells with rosiglitazone (1 μM, 24 h) and subsequently assessed dose-response to vincristine. Rosiglitazone pretreatment enhanced vincristine sensitivity, as shown by a shift in dose-response curves (Figure 6G) and significantly reduced viability at representative concentrations (Figure 6H). The effect was reproducible in an independent resistant line, PANC-1, confirming broader applicability (Figures 6I and 6J). Pretreatment with rosiglitazone also modestly improved responses to carboplatin and paclitaxel, though with variable magnitude across cell lines (Figures S5E–S5H). Collectively, these results provide proof-of-principle evidence that the pharmacologic suppression of NFE2L2-linked transcriptional programs, particularly by rosiglitazone, can partially re-sensitize drug-resistant cancer cells, reinforcing the predictive validity of our computational screening pipeline.

## Discussion

Overcoming broad drug resistance in cancer remains a significant challenge, requiring strategies that address the multifactorial nature of therapeutic failure across diverse cancer types. Here, we developed and applied an integrative computational framework to systematically characterize broad drug resistance in cancer cell lines, connecting drug-response phenotypes to multi-omic profiles, transcriptional networks, and potential re-sensitization strategies using perturbagens.

Using PRISM repurposing data from DepMap, we established a composite drug-sensitivity score across mechanistically diverse compounds to stratify cell lines as broadly sensitive or resistant. This approach enabled the identification of consistent drug-response phenotypes independent of individual compound or target classes, providing a foundation for subsequent omics analyses. By incorporating multiple strategies to define resistant and sensitive groups, including direct sensitivity score stratification, clustering to reduce intra-group heterogeneity, and lineage-balanced grouping, we ensured that identified features were robust and not artifacts of specific group definitions. This multi-angle approach allowed us to dissect the underlying biology of broad resistance while minimizing potential biases related to lineage or dataset-specific confounders.^24^^,^^47^^,^^48^^,^^49^

Transcriptomic analyses revealed a convergence on gene programs associated with extracellular matrix (ECM) remodeling, stress response, and survival signaling, with NFE2L2 (NRF2) emerging as a key transcriptional regulator upregulated in resistant lines. Pathway analyses further highlighted TGF-β signaling, IGF transport, and metabolic reprogramming, including amino acid and nucleotide biosynthesis, as hallmarks of resistant phenotypes. This multi-omic convergence was reinforced by metabolomics integration, where differentially abundant metabolites and DEGs were enriched in shared pathways, underscoring the rewiring of metabolic dependencies in resistant states.

Although two of the three grouping strategies yielded FDR-significant DEGs, the lineage-balanced analysis did not, despite consistent nominal significance (*p* < 0.05). This likely reflects the smaller effective sample size and reduced statistical power when balancing across diverse tissue lineages, where expression variance is higher. Nevertheless, the concordance of direction and overlap across all three strategies suggests that the identified programs, particularly those involving ECM remodeling and NFE2L2 signaling, represent core determinants of broad resistance rather than lineage-restricted phenomena.

Several metadata features were also significantly associated with the composite sensitivity score after FDR correction (23/507, q < 0.05), most of which corresponded to Oncotree primary disease classifications rather than broader contextual variables such as lineage, subtype, or collection site. Because disease-type annotations often correlate with lineage, our lineage-balanced analysis (Group 3) partially controls for these effects and yielded 585 DEGs that overlap with DEGs from our other grouping strategies. These findings indicate that the observed transcriptional programs are not driven by dataset-level or lineage-specific biases but instead reflect shared resistance mechanisms across cancer contexts. Despite these controls on lineage-biases, limitations to this study remain. Future studies leveraging lineage-specific or single-cell-resolved transcriptomic analyses will be essential to dissect context-dependent components of this program that may be masked when aggregating across heterogeneous cancer types. In particular, the application of similar integrative frameworks to pediatric tumor datasets, such as the Childhood Cancer Model Atlas (CCMA), will enable refined, context-aware analyses that better capture disease- and lineage-specific resistance programs.

Our mutation analyses, employing both gene-level frequency comparisons and pathway-level burden analyses, revealed distinct but complementary insights. While gene-level comparisons identified pathways associated with chromatin remodeling and stress response in sensitive lines and growth factor signaling in resistant lines, pathway-level analyses across all mutations captured broader patterns of pathway enrichment, including ECM-related processes and signaling pathways relevant to resistance. Notably, the observation that Glycosaminoglycan degradation and ECM proteoglycans were enriched but directionally discordant across analysis methods emphasizes the importance of granularity in data interpretation and the complex role of ECM dynamics in modulating drug response.

Constructing an integrative gene-regulator network combining DEGs, recurrent mutations, and enriched transcription factors revealed a network of functional convergence in resistant lines. NFE2L2, a well-established regulator of oxidative stress responses and xenobiotic detoxification, previously implicated in chemoresistance across multiple cancer types,^50^^,^^51^^,^^52^^,^^53^^,^^54^^,^^55^ emerged as a central hub. It is connected to upstream mutations in genes such as KEAP1, EP300, ERBB4, TCF12, SMC1A, and NIPBL, and to downstream transcriptional programs involved in oxidative stress response and survival. Several of these genes, including KEAP1^56^^,^^57^^,^^58^ and EP300,^59^^,^^60^^,^^61^ are well-known cancer drivers or regulators of NFE2L2 activity, and targeting this axis has recently been implicated in re-sensitization strategies.^62^ Others, such as TCF12 and NIPBL, are less commonly associated with cancer or drug resistance; TCF12 is classified as a lower-confidence cancer gene in OncoKB, and NIPBL is not listed, suggesting potential novel roles in mediating transcriptional reprogramming. The tendency of these mutations to occur in a largely non-overlapping pattern while collectively impacting NFE2L2 function supports a model of convergent evolution toward a resistant phenotype through diverse genomic alterations.

Extending these findings to patient cohorts using cBioPortal and TCGA PanCancer data demonstrated that the mutation signatures identified in resistant cell lines were also prevalent in patient tumors across multiple cancer types, with strong representation in cancers known for treatment challenges, including lung and endometrial carcinomas. Importantly, patients harboring these resistance-associated mutations exhibited significantly shorter progression-free survival, supporting the translational relevance of these molecular features in mediating therapeutic resistance in human cancers.

Building on this integrative characterization, we employed resistance-associated DEGs to computationally screen perturbagen libraries, including LINCS, Drug Atlas, and DRUGseq datasets, to identify compounds capable of reversing resistance-associated transcriptional profiles. This approach leverages large-scale perturbagen datasets generated and characterized in foundational studies,^24^^,^^42^^,^^44^^,^^45^ enabling the systematic exploration of resistance reversal strategies. Recent advancements in perturbagen screening at high resolution, including multiplex single-cell RNA-Seq pharmacotranscriptomics^40^ and TraCe-seq clonal fitness profiling,^41^ have highlighted the potential of transcriptional profiling to dissect drug response heterogeneity. While our study did not employ single-cell approaches, it complements these efforts by integrating bulk multi-omic data with perturbagen signatures to systematically prioritize candidate compounds for re-sensitization. This rational screening approach favors compounds predicted to repress genes overexpressed in resistant lines while reactivating suppressed genes, guiding drug combination selection beyond empirical methods. These findings align with broader efforts to map drug response and resistance mechanisms at scale, as exemplified by cancer dependency mapping^23^ and multi-omic functional target identification.^47^^,^^63^ Drug-gene network analyses revealed convergence on stress regulators, drug metabolism genes, and efflux transporters such as NFE2L2, ABCB1, CYP3A4, and EGFR, highlighting actionable targets for re-sensitization. Focused analyses of NFE2L2-centered networks identified compounds such as halcinonide with high overlap between DEGs high in resistant lines, genes predicted to be downregulated by the perturbagen, and NFE2L2 transcriptional targets, illustrating actionable strategies for disrupting resistance pathways.

Integration with DepMap PRISM drug response data demonstrated that candidate compounds such as brefeldin A and nocodazole exhibit significant activity in resistant lines, indicating target engagement and the potential to reprogram resistant cells toward a sensitive phenotype. Alternatively, compounds that reprogram resistance-associated transcriptional signatures without impacting cell viability, that could offer selective therapeutic advantages by minimizing toxicity to normal tissues, may be worth further investigating.

Lastly, to experimentally validate our computational predictions, we tested representative compounds predicted to repress NFE2L2-associated resistance programs using transcriptomic and functional assays. Short-term treatment (1 μM, 24 h) with halcinonide, methylprednisolone, and rosiglitazone in the broadly resistant OE19 cell line induced clear transcriptional divergence from vehicle controls and downregulation of canonical NFE2L2 target genes, consistent with predicted pathway reversal. Among these, rosiglitazone produced the most pronounced transcriptional modulation, reducing the enrichment of oxidative-stress and detoxification pathways while increasing cell-cycle and DNA-repair gene expression. Functionally, brief pretreatment with rosiglitazone partially re-sensitized resistant cells to multiple chemotherapeutics, in both OE19 and an independent resistant line, PANC-1, demonstrating reproducibility across lineages. These results provide proof-of-principle that the computational framework can successfully prioritize perturbagens capable of functionally reprogramming resistant phenotypes through transcriptional network reversal. However, further functional validation of prioritized compounds in resistant models, including transcriptomic profiling post-treatment and preclinical testing in combinations with standard-of-care therapies, will be essential to translate these predictions into effective strategies to overcome broad drug resistance in cancer.

### Limitations of the study

While this study presents a scalable computational framework for integrating multi-omics datasets with perturbagen signatures to rationally identify candidate compounds for reversing broad drug resistance, several limitations should be acknowledged. Our analyses relied on large-scale screening datasets that may not fully capture the complexity of *in vivo* tumor microenvironments or resistance mechanisms arising under clinical treatment pressures. Moreover, because our analyses are based on bulk cell lines and multi-omic datasets, they do not capture the cellular and microenvironmental heterogeneity that shapes drug response in tumors. *In vivo* resistance is often driven by non-cell-autonomous mechanisms, including paracrine signaling from stromal fibroblasts, immune-mediated feedback, hypoxia, and nutrient gradients within the tumor microenvironment. These factors can alter pathway activation (e.g., NFE2L2, TGF-β, or ECM remodeling programs) in ways that are not reflected in homogenous culture models. Likewise, bulk transcriptomic averaging masks cell-state diversity, potentially obscuring subpopulations that are transiently drug-tolerant or primed for resistance. Incorporating single-cell and spatial transcriptomic approaches, as well as co-culture or organoid systems that model tumor-microenvironment interactions, will be essential to resolve these context-dependent dynamics. Future extensions of this computational framework could integrate such data to model cell-cell interactions and adaptive reprogramming, yielding a more complete systems-level understanding of broad drug resistance.

Emerging single-cell perturbation approaches, such as those demonstrated in recent high-throughput pharmacotranscriptomic pipelines^40^ and TraCe-seq,^41^ offer promising avenues to address these challenges by capturing intra-tumor heterogeneity and adaptive responses. Moreover, while we focused on NFE2L2 as a central regulator linking transcriptional reprogramming, stress response, and multidrug resistance, other pathways identified in our analyses warrant further exploration. For example, glycosaminoglycan metabolism and glycosylation-related pathways frequently emerged in enrichment analyses (Tables S7, S8, S9, S10, S11, S12, and S13) but were not prioritized here. Future studies should evaluate how these pathways contribute to ECM remodeling, signaling, and resistance phenotypes.

## Resource availability

### Lead contact

Further information and requests for resources should be directed to and will be fulfilled by the lead contact, Dr. Aktar Ali (aali4@nd.edu).

### Materials availability

This study did not generate new unique reagents.

### Data and code availability

• All omics and drug screening datasets analyzed in this study are publicly available from the DepMap portal (https://depmap.org) and for the analyses of clinical samples from multiple patient cohorts in cBioPortal (https://cbioportal.org). The combined study contains samples from the following 32 studies: Acute Myeloid Leukemia (TCGA, PanCancer Atlas), Adrenocortical Carcinoma (TCGA, PanCancer Atlas), Bladder Urothelial Carcinoma (TCGA, PanCancer Atlas), Brain Lower Grade Glioma (TCGA, PanCancer Atlas), Breast Invasive Carcinoma (TCGA, PanCancer Atlas), Cervical Squamous Cell Carcinoma (TCGA, PanCancer Atlas), Cholangiocarcinoma (TCGA, PanCancer Atlas), Colorectal Adenocarcinoma (TCGA, PanCancer Atlas), Diffuse Large B-Cell Lymphoma (TCGA, PanCancer Atlas), Esophageal Adenocarcinoma (TCGA, PanCancer Atlas), Glioblastoma Multiforme (TCGA, PanCancer Atlas), Head and Neck Squamous Cell Carcinoma (TCGA, PanCancer Atlas), Kidney Chromophobe (TCGA, PanCancer Atlas), Kidney Renal Clear Cell Carcinoma (TCGA, PanCancer Atlas), Kidney Renal Papillary Cell Carcinoma (TCGA, PanCancer Atlas), Liver Hepatocellular Carcinoma (TCGA, PanCancer Atlas), Lung Adenocarcinoma (TCGA, PanCancer Atlas), Lung Squamous Cell Carcinoma (TCGA, PanCancer Atlas), Mesothelioma (TCGA, PanCancer Atlas), Ovarian Serous Cystadenocarcinoma (TCGA, PanCancer Atlas), Pancreatic Adenocarcinoma (TCGA, PanCancer Atlas), Pheochromocytoma and Paraganglioma (TCGA, PanCancer Atlas), Prostate Adenocarcinoma (TCGA, PanCancer Atlas), Sarcoma (TCGA, PanCancer Atlas), Skin Cutaneous Melanoma (TCGA, PanCancer Atlas), Stomach Adenocarcinoma (TCGA, PanCancer Atlas), Testicular Germ Cell Tumors (TCGA, PanCancer Atlas), Thymoma (TCGA, PanCancer Atlas), Thyroid Carcinoma (TCGA, PanCancer Atlas), Uterine Carcinosarcoma (TCGA, PanCancer Atlas), Uterine Corpus Endometrial Carcinoma (TCGA, PanCancer Atlas), Uveal Melanoma (TCGA, PanCancer Atlas).
• Processed data and custom analyses performed using the DepMap portal used to generate figures and perform pathway/network analyses available in supplemental tables. No novel code was used for analyses or the generation of figures. Any additional information required to reanalyze the data reported in this article is available from the lead contact upon request.
• The perturbation validation RNA-seq data discussed in this publication have been deposited in NCBI’s Gene Expression Omnibus^64^ and are accessible through GEO Series accession number GSE309935.
• Additional supporting data are available upon request from the lead contact.

## Acknowledgments

This work was supported by funding from the Warren Family Research Center for Drug Discovery and Development. We also acknowledge the University of Notre Dame Biological Screening and Development Core for resources and the University of Notre Dame Genomics and Bioinformatics Core Facility for providing sequencing support. The project benefited from GBCF services, including nucleic acid quality control, library preparation, sequencing, and data transfer. We thank the GBCF staff for their technical expertise and infrastructure, which were essential for the completion of this work.

## Author contributions

I.M.: Conceptualization, methodology, investigation, formal analysis, data curation, visualization, and writing – original draft. B.B.: Supervision, project administration, and funding acquisition. A.A.: Conceptualization, supervision, funding acquisition, and writing – review and editing.

## Declaration of interests

The authors declare no competing interests.

## Declaration of generative AI and AI-assisted technologies in the writing process

During the preparation of this work, the authors used OpenAI’s ChatGPT to assist with editing and language refinement. After using this tool, the authors reviewed and edited the content as needed and take full responsibility for the content of the publication.

## STAR★Methods

### Key resources table

**Table:** 

REAGENT or RESOURCE	SOURCE	IDENTIFIER
**Chemicals, peptides, and recombinant proteins**		

Rosiglitazone	Selleckchem	Cat# S2556
Halcinonide	Selleckchem	Cat# S4098
Methylprednisolone	Selleckchem	Cat# S1733
Paclitaxel	Selleckchem	Cat# S1156
Vincristine	Selleckchem	Cat# S1261
Carboplatin	MedChem Express	Cat# HY-17393

**Critical commercial assays**		

Cell Titer-Glo® 2.0 Assay	Promega	Cat# G9243
RNeasy Plus Mini Kit	Qiagen	Cat# 74134

**Deposited data**		

Drug screening data, transcriptomics, mutation data (25Q2)	Broad Institute	https://www.depmap.org/portal
TCGA, PanCancer Atlas	Cbioportal	https://www.cbioportal.org/
RNA-seq data, perturbagen validation experiments, OE19	This paper	GSE309935

**Experimental models: Cell lines**		

OE19 cell line, human (male)	Sigma Aldrich	Cat# 96071721-1VL; RRID: CVCL_1622
PANC-1 cell line, human (male)	ATCC	Cat# CRL-1469; RRID: CVCL_0480

**Software and algorithms**		

Cytoscape (v3.9.1)	Shannon et al.^68^	https://cytoscape.org
DESeq2 (v1.42.0)	Love et al.^76^	https://bioconductor.org/packages/DESeq2
DGIdb 4.2	Freshour et al., 2021	https://www.dgidb.org
DrugEnrichr	Kuleshov et al.^72^	https://maayanlab.cloud/DrugEnrichr
Enrichr	Kuleshov et al., 2016	https://maayanlab.cloud/Enrichr
featureCounts (v2.0.1)	Liao et al.^75^	http://bioinf.wehi.edu.au/featureCounts/
GraphPad Prism (v10)	GraphPad Software	https://www.graphpad.com
HISAT2 (v2.2.1)	Kim et al.^74^	https://daehwankimlab.github.io/hisat2/
MetaboAnalyst 5.0	Xia et al., 2009	https://www.metaboanalyst.ca
R (v4.3.1)	R Core Team	https://cran.r-project.org
SigCom LINCS	Evangelista et al.^42^	https://maayanlab.cloud/sigcom-lincs

### Experimental model and study participant details

#### Cell lines and culture conditions

All experiments were conducted using authenticated human cancer cell lines obtained from Sigma Aldrich (OE19) or ATCC (PANC-1).

OE19: esophageal adenocarcinoma; male sex; RRID: CVCL_1622.

PANC-1: pancreatic ductal adenocarcinoma; male sex; RRID: CVCL_0480.

Cells were maintained in DMEM/F12 supplemented with 10% FBS and 1% penicillin–streptomycin at 37 °C in 5% CO_2_. Cells were regularly tested for mycoplasma contamination and used within limited passages. This study used only commercially available, previously established human cancer cell lines. No animal or human subjects were used. All cell lines were obtained from certified repositories, authenticated by the suppliers, and are exempt from IRB oversight. Both cell lines used in this study are of male origin. The influence of sex on the study results cannot be determined because no female-derived models were available for experimental validation. This is noted as a limitation regarding generalizability.

### Method details

#### Drug sensitivity data and calculation of global resistance scores

Drug sensitivity data were obtained from the PRISM Repurposing dataset^24^ (DepMap 24Q2), containing log_2_ fold-change (LFC) viability scores across 6765 compounds in cancer cell lines. For each cell line, compound responses from PRISM Repurposing data were grouped by mechanism of action (MoA). Class-level median log_2_ fold changes were computed and averaged to generate a composite sensitivity score, giving equal weight to each drug class. This approach mitigates biases due to uneven representation of compound classes in PRISM (ex. more kinase inhibitors than epigenetic modulators) and ensures balanced assessment of broad drug-response behavior.

#### Selection and refinement of resistant and sensitive cell lines

Cell lines were grouped into sensitive and resistant populations using 3 different strategies.

(1) Cell lines in the top and bottom deciles of drug sensitivity scores were classified as resistant or sensitive, respectively.
(2) To reduce intra-group heterogeneity, a supervised feature selection was performed using correlation analysis (expression vs. sensitivity score) followed by ANOVA F-test to retain the top 250 discriminatory genes. Z-score normalization was applied, and hierarchical clustering (Ward’s method, Euclidean distance) was performed to identify the largest contiguous, phenotype-pure clusters for downstream analyses.
(3) Lineage balanced groups were constructed to control for lineage-specific biases by including only cancer types represented in both resistant and sensitive categories (top 10%) and selecting an equal number of a specific lineage based on highest or lowest drug sensitivity score for resistant and sensitive lines respectively.

#### Metadata association analyses

We tested associations between the composite drug-sensitivity score and DepMap metadata (growth pattern, onboarded media, primary disease, age, sample collection site, etc.). Associations were evaluated by univariate models and adjusted for multiple testing using the Benjamini–Hochberg procedure across all features (m = 507). In total, 23 features remained significant at FDR q < 0.05.

#### Differential gene expression and metabolomics analysis

Differential gene expression analyses were performed using the DepMap Custom Analysis tool (https://depmap.org/portal/interactive/custom_analysis), conducting two-class comparisons between resistant and sensitive cell-line subsets defined by composite drug-sensitivity scores. This tool computes effect sizes (interpreted as fold differences between groups) and P values on RNA-seq gene-expression data reported as log_2_(TPM + 1) for each gene across cell lines. For exploratory analyses and to capture subtle differences between sensitive and resistant populations, all genes with P < 0.05 were initially considered significant. To increase robustness, we further filtered these candidates by performing a correlation analysis between drug-sensitivity score and gene expression (log_2_[TPM + 1] values from DepMap). Correlations were retained if | r | > 0.15 with q < 0.05 (Benjamini–Hochberg correction).

Genes identified as differentially expressed in all three of the independent grouping strategies (top/bottom deciles, cluster-based, and lineage-balanced subsets) were compiled for downstream analyses. Across these comparisons, 585 (509 upregulated and 76 downregulated) genes were consistently differentially expressed (P < 0.05) between resistant and sensitive groups. Among these, all 585 had q < 0.05 in Group 1, 563 of 585 had q < 0.05 in Group 2, and none reached q < 0.05 in Group 3 (lineage balanced), although all retained nominal P < 0.05. DEGs were further filtered for indicated exploratory analyses including effect size cutoff of >1 for enrichment analyses.

Differential metabolite-abundance analyses were conducted in the same manner using the DepMap Custom Analysis tool, comparing resistant and sensitive groups based on LC-MS metabolomics data. All P values were adjusted for multiple testing using the Benjamini–Hochberg false-discovery rate (FDR), and both raw and adjusted values are reported in Tables S5, S6, and S9.

#### Pathway and transcription factor enrichment analysis

Pathway enrichment analyses were performed using Enrichr^65^ (https://maayanlab.cloud/Enrichr/), querying Reactome and ENCODE/ChEA consensus transcription factor databases on upregulated and downregulated DEGs. Results are reported as nominal P-values unless otherwise indicated and -log10(P) transformed for bar graph/volcano plot visualization; enrichments meeting FDR-adjusted P < 0.05 are specifically marked as significant.

#### Metabolomics data integration and joint pathway analysis

Metabolomics data were integrated using differentially abundant metabolites from DepMap. Combined DEG and metabolite sets were analyzed in MetaboAnalyst 5.0^66^ (https://www.metaboanalyst.ca). Statistical significance for pathway enrichment was determined at P < 0.05, with FDR-adjusted P < 0.05 considered significant where indicated. Pathway impact values were calculated using topology-based metrics within MetaboAnalyst.

#### Mutation analysis and pathway-level burden scoring

Binary gene-level mutation data were obtained from the DepMap database. Because mutation data were not available for all cell lines included in the gene-expression analyses, we restricted this analysis to those lines for which both mutation and drug-sensitivity data were available within the top 10% most resistant and most sensitive groups (n = 91 per group in the transcriptomic analysis; n ≈ 50 per group with complete mutation data). This ensured consistent representation of the most phenotypically extreme lines while maximizing available sample size.

Gene-level mutation frequency skew scores (resistant vs. sensitive) were computed to identify genes with differential mutation burdens. The top 50 skewed genes were subjected to functional enrichment analysis using Enrichr, with statistical significance determined by Fisher’s exact test and Benjamini–Hochberg FDR correction (adjusted P < 0.05).

For pathway-level analyses, sample-wise mutation burden scores were calculated using Reactome gene sets. For each individual sample, enrichment was computed as –log_10_(P-value), and group-level pathway impact was defined as:

$$\text{Pathway}\;\text{impact}=\left(\%\;\text{of}\;\text{lines}\;\text{significantly}\;\text{enriched}\right)\times \left(\text{mean}-\log_{10}\left(P-\text{value}\right)\;\text{per}\;\text{group}\right)$$

Group skew was calculated as the difference in pathway impact between resistant and sensitive groups. Heatmaps and volcano plots were generated to visualize differential pathway-level mutation burdens and highlight pathways enriched in resistant populations.

#### Network construction and visualization

Gene–regulator networks were constructed by integrating protein-protein interactions (PPIs) as edges from STRING^67^ (high confidence, direct physical interactions) between DEGs and genes with mutations (nodes), as well as transcription factors – target regulatory interactions between genes from ENCODE/ChEA, using Cytoscape 3.9.1^68^ (https://cytoscape.org) for visualization. Focused subnetworks centered on NFE2L2 and associated modules were generated for pathway-centric analyses.

#### Patient cohort analyses in cBioPortal

Clinical relevance was assessed using cBioPortal^69^ (https://www.cbioportal.org), querying TCGA PanCancer Atlas^70^^,^^71^ data for mutations in KEAP1, EP300, ERBB4, TCF12, SMC1A, and NIPBL across 32 cancer types. Mutation frequencies, co-occurrence analyses, and Kaplan-Meier survival plots (log-rank test) for overall and progression-free survival were generated using integrated cBioPortal tools.

#### Perturbagen screening and drug–gene network analyses

##### Perturbagen and drug mechanism enrichment analyses

Resistance-associated DEGs (P < 0.05) were used to query LINCS L1000, NIBR DRUGseq 2025, and Proteomics Drug Atlas 2023 perturbation libraries via the SigCom LINCS platform^42^ (https://maayanlab.cloud/sigcom-lincs) and EnrichR. The top 100 significantly enriched perturbagens were retained from each library, all meeting Benjamini–Hochberg adjusted significance thresholds (FDR-adjusted P < 0.05). Enrichment for drug mechanisms of action was performed using DrugEnrichr^72^ (https://maayanlab.cloud/DrugEnrichr). Drug–gene interactions were mapped using DGIdb 4.2^73^ (https://www.dgidb.org), and networks were visualized in Cytoscape.

#### Predicted compound activity validation using DepMap PRISM

Candidate compound activities were evaluated by extracting PRISM repurposing dataset cell viability data (log_2_ fold change) in resistant and sensitive lines to confirm bioactivity profiles and prioritize compounds for re-sensitization potential.

#### RNA-seq perturbation profiling

##### Cell culture and treatments

OE19 cells were exposed for 24 h to halcinonide, methylprednisolone, or rosiglitazone (1 μM each; 0.1% DMSO vehicle for controls). Each condition included n = 2 biological replicates.

##### RNA extraction, sequencing, and preprocessing

Total RNA was isolated using the Qiagen RNeasy Mini Kit per manufacturer’s instructions. Libraries were prepared by the Notre Dame Genomics Core and sequenced on an Illumina NextSeq 2000 (paired-end, PE50), targeting ∼30 million reads/sample. FASTQ files were aligned to GRCh38/hg38 with HISAT2^74^; gene-level counts were generated with featureCounts.^75^

Normalization, QC, and dimensionality reduction. Count matrices were analyzed with DESeq2.^76^ Size factors and dispersions were estimated using the default pipeline; low-abundance genes were filtered by DESeq2’s independent filtering. For unsupervised analyses, counts were variance-stabilized (VST) and used to compute (i) PCA and (ii) sample-to-sample Euclidean distances (all treatment conditions vs. controls).

##### Differential expression analyses

DESeq2 analysis was performed for each compound separately vs. DMSO control. Unless otherwise indicated, significance required P < 0.05 with an effect-size filter of log_2_FC < -0.5.

Enrichr used for enrichment analyses performed using DEGs downregulated (log_2_FC < -0.5) after treatment. Enrichment P-values were computed and FDR-adjusted P < 0.05 was considered significant (reported as FDR where indicated).

Overlap statistics with resistance markers. Overlap between genes up in resistant lines and genes down after perturbation was tested using two-sided Fisher’s exact tests with a background of 27,771 expressed genes (RNA-seq universe). Where multiple overlaps were tested (across drugs/cutoffs), P-values were BH-adjusted; we report odds ratios and (adjusted) P-values in Table S15.

Reproducibility and reporting. All thresholds used in Figure 6 are indicated on the corresponding legends/panels: primary calls at FDR q < 0.05 (or 0.10 where noted) and |log_2_FC| ≥ 0.5, with exploratory Venn analyses additionally shown at P < 0.05 & |log_2_FC| ≥ 0.5/1.0.

Data availability. Raw and processed RNA-seq data are currently hosted on usegalaxy (temporary access during the U.S. government shutdown). GEO accession will be provided and linked in the manuscript upon repository reopening.

#### Cell-viability assays and perturbagen testing

OE19 and PANC-1 cells were pretreated for 24 h with 1 μM rosiglitazone or 0.1% DMSO vehicle in 6-well plates under standard culture conditions. After pretreatment, cells were harvested, counted, and re-plated into 96-well plates (3 × 10^3^ cells/well) for dose-response testing with standard chemotherapeutic agents (vincristine, carboplatin, and paclitaxel). After 72 h of exposure, cell viability was quantified using the CellTiter-Glo luminescent assay (Promega) according to manufacturer’s instructions. Luminescence values were background-subtracted and normalized to within-plate vehicle controls. Dose-response curves were generated in GraphPad Prism, and normalized viability values were plotted as mean ± SD of 4 biological replicates. Statistical significance was evaluated only at the specified concentrations shown in the bar-graph panels using multiple unpaired two-tailed t-tests, with Benjamini–Hochberg (FDR) correction. Adjusted P < 0.05 was considered significant.

### Quantification and statistical analysis

All statistical analyses were performed using the DepMap Custom Analysis Portal, R (v4.3), GraphPad Prism (v10), Excel, Cytoscape v3.9.1, Enrichr, DrugEnrichr, SigCom LINCS tools, and MetaboAnalyst 5.0. Exact tests, n values, statistical parameters, and significance thresholds are reported in the figure legends and Tables S4, S5, S6, S7, S8, S9, S10, S11, S12, S13, S14, and S15.

#### RNA-seq analyses

DEGs determined through DepMap custom analyses (two-class comparisons). Significance: q < 0.05 for primary analyses; exploratory analyses used nominal P < 0.05 where indicated. Effect-size filters: |log_2_FC| ≥ 0.5 or 1.0 as described in each figure. For correlation analyses/Pearson correlation coefficient determined between expression (log_2_[TPM+1]) and drug-sensitivity score; significance defined as q < 0.05 and |r| > 0.15.

#### For perturbagen validation RNA-seq

n = 2 biological replicates per condition. DESeq2 with default shrinkage; significance P < 0.05 and |log_2_FC| < −0.5 unless otherwise indicated. For downstream enrichment analysis, significance threshold set at FDR < 0.05.

#### Metabolomics

Differential metabolite abundance was assessed via DepMap LC-MS metabolomics data analyzed by two-class comparison (DepMap custom analysis tool). Significant metabolites defined as FDR < 0.05 (primary) or P < 0.05 (exploratory). Combined DEG + metabolite pathway impact scores computed via topology-based metrics in MetaboAnalyst.

#### Enrichment and pathway analyses

For all enrichment analyses using Enrichr (Reactome, ENCODE/ChEA), DrugEnrichr, MetaboAnalyst, PANGEA, or SigCom LINCS, enrichment significance: nominal P < 0.05 or FDR < 0.05 where specified.

#### Mutation analyses

For gene-level mutation analysis, frequency differences between resistant and sensitive groups were analyzed using skew scores defined as (% mutated resistant − % mutated sensitive), top 50 by skew score used for downstream enrichment analysis.

For pathway level analyses, pathway impact scores calculated as (% enriched lines) × (mean –log_10_P) and group skew was calculated as the difference in pathway impact between resistant and sensitive groups. Threshold of P < 0.05 considered significant for enrichment analyses of individual lines.

#### Network analyses

Cytoscape networks constructed using protein–protein interaction networks from STRING (high confidence, direct interactions), TF–target networks (ENCODE/ChEA regulatory databases), and drug-gene interactions from DGIDB. Network topology was descriptive and not statistically modeled.

#### Perturbagen signature analyses

Resistance-associated DEGs were used to query perturbation libraries (LINCS L1000, DRUGseq, Drug Atlas).

Enrichment significance: BH-FDR < 0.05. Overlap between resistant-upregulated genes and perturbagen-downregulated genes assessed by two-sided Fisher’s exact tests with background gene universe: 27,771 expressed genes.

#### Cell viability assays

Cell viability following drug exposure was quantified using CellTiter-Glo (n = 4 biological replicates). Data presented as mean ± SD. Dose–response curves fitted in Prism. Statistical tests for specified concentrations (bar-graph comparisons) used multiple unpaired two-tailed t-tests with Benjamini–Hochberg FDR correction. Adjusted P < 0.05 considered significant.

#### General statistical considerations

Assumptions of normality were not formally tested; parametric tests are acceptable given sample size and conventional usage for viability assays. No outlier removal was performed. Samples were not randomized because all experiments used pre-established cancer cell lines assigned to treatment groups based on experimental design. Blinding was not relevant to this study because all measurements (cell viability, RNA-seq quantification, and computational analyses) were generated by automated assays or software pipelines without subjective scoring steps. All thresholds used for statistical significance are directly stated in figure legends.

Full statistics, including exact P values, effect sizes, confidence intervals, DESeq2 outputs, and enrichment values, are provided in Tables S1, S2, S3, S4, S5, S6, S7, S8, S9, S10, S11, S12, S13, S14, and S15.

## Supplemental information

Document S1. Figures S1–S5

Table S1. Depmap cell lines and median drug sensitivity scores for separate drug classes and rank-based variance analysis

Table S2. Cell lines and associated metadata for sensitive and resistant analysis groups

Table S3. Cell line metadata features used for univariate analysis

Table S4. Univariate analysis of metadata features

Table S5. Depmap custom analyses (two class comparisons) comparing gene expression in sensitive and resistant lines from our 3 separate grouping strategies, and correlation analysis plotting gene expression vs. drug sensitivity score (all lines)

Table S6. Overlapping DEGs from separate grouping strategies AND significant correlation between drug sensitivity score and gene expression

Table S7. Transcription factor enrichment analysis from EnrichR with ENCODE and ChEA consensus TFs gene set library

Table S8. EnrichR enrichment analysis for DEGs (shared p < 0.05 from 2-class comparisons and q < 0.05 from correlation analysis)

Table S9. Depmap custom analyses (two class comparisons) comparing metabolomics data in sensitive and resistant lines from our 3 separate grouping strategies, and correlation analysis plotting metabolite abundance vs. drug sensitivity score (all lines)

Table S10. Results from Metaboanalyst joint pathway analysis using DEGs and metabolites from Tables S6 and S9

Table S11. Multiple functional enrichment analyses using PANGEA with top 50 mutations from each group

Table S12. Separate pathway enrichment analyses using all damaging mutations for each individual cell line

Table S13. Top 100 perturbagens predicted to reverse RESISTANT gene expression signatures from 4 separate databases or gene set libraries

Table S14. NFE2L2 interactors from DGIDB and predicted perturbagen and overlap of predicted perturbagen DEGs and NFE2L2 transcriptional targets

Table S15. Perturbagen validation with overlap statistics using DEGs from indicated compound in OE19 cells and resistance associated genes
