# Supplementary material for: Multi-omic integration identifies broad drug resistance mechanisms and strategies to therapeutically reprogram cancer cells
Source: iScience. 2025 Nov 28;29(1):114293. doi: 10.1016/j.isci.2025.114293 (PMC12765193; doi:10.1016/j.isci.2025.114293)
Supplement: Document S1. Figures S1–S5 [file mmc1.pdf]

iScience, Volume *29*

Supplemental information

Multi-omic integration identifies broad drug resistance mechanisms and strategies to therapeutically reprogram cancer cells

Ian Mersich, Brian S.J. Blagg, and Aktar Ali

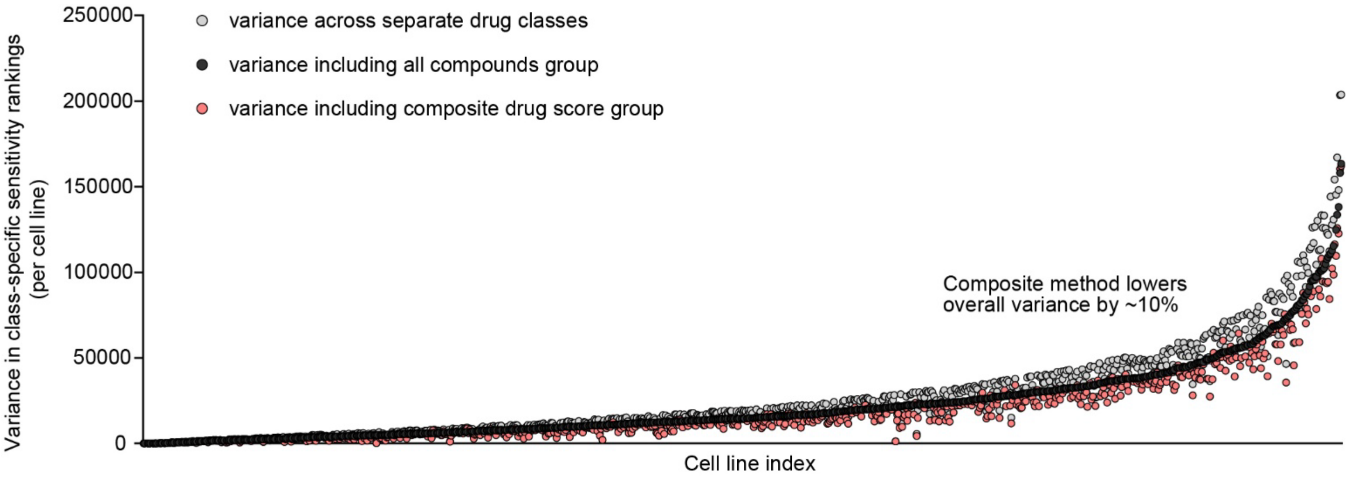

Supplemental Figure S1 (related to Figure 1). Comparison of variance across rank-based drug-class sensitivity scores.

A rank based method was used to score cell lines for individual drug classes. Variance in class-specific drug-sensitivity rankings is shown for each DepMap cell line (x-axis, ordered by variance). Grey points represent variance computed across ranks of individual drug classes only; black points include variance incorporating the all-compounds median LFC rank; and red points include variance incorporating the composite drug-score rank. The composite method consistently yields lower variance across cell lines, corresponding to an approximate 10 % reduction in total variance (from 20.5×10^6 to 18.3×10^6), indicating improved stability and generalizability of the composite drug-sensitivity score over a simplified median LFC for all compounds scoring method.

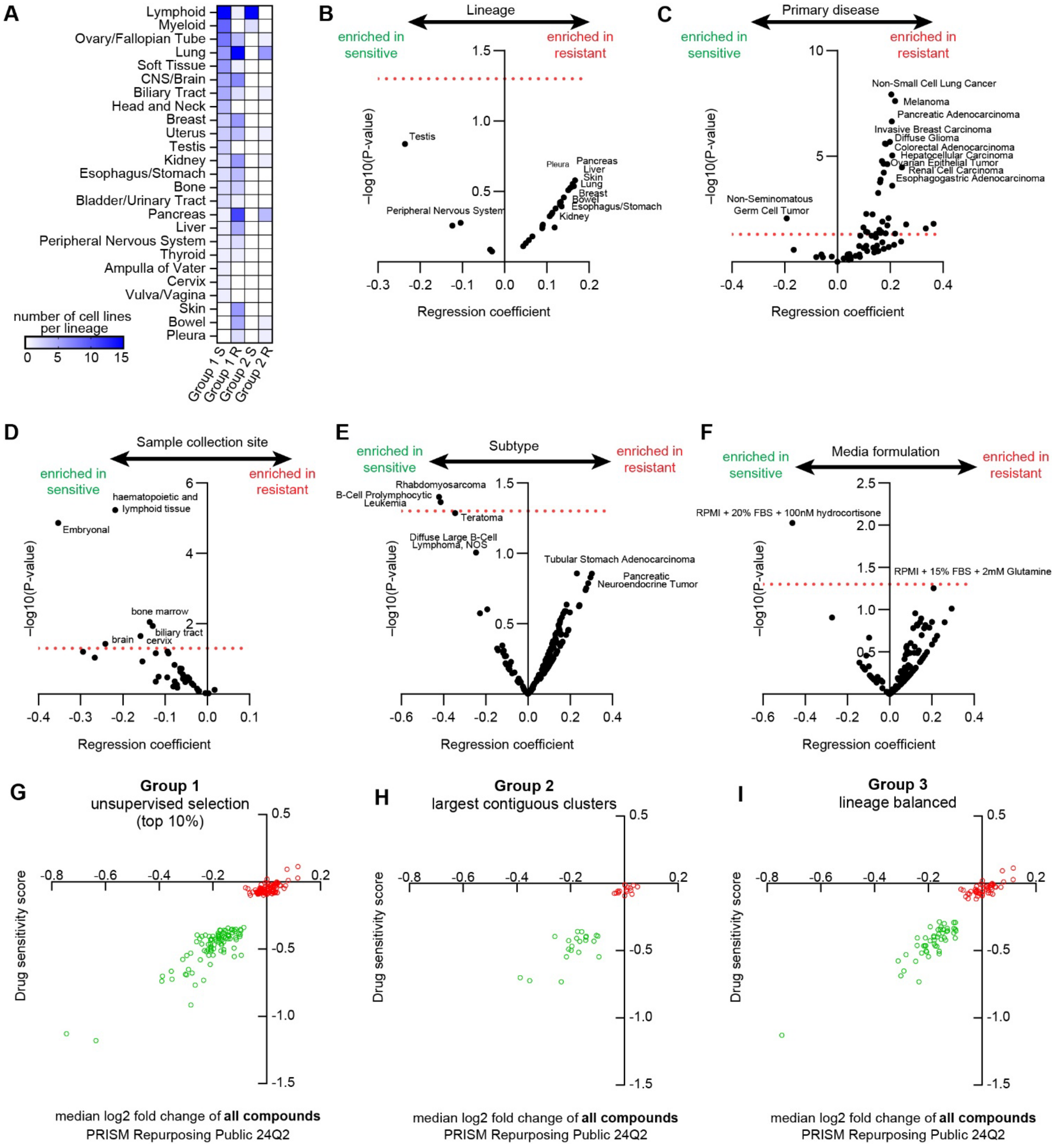

Supplemental Figure S2 (related to Figure 2). Additional stratification, metadata, and drug sensitivity analyses for resistant and sensitive group definitions.

(A) Lineage distribution across resistant and sensitive groups reveals imbalances in Groups 1 and 2.
(B) Regression analysis of lineage versus drug sensitivity scores shows no significant associations.
(C–F) Assessment of additional metadata (sample collection site, primary disease, subtype, media formulation) for potential confounding effects.
(G–I) Drug sensitivity plots confirming clear stratification between resistant and sensitive populations across all three comparison groups.

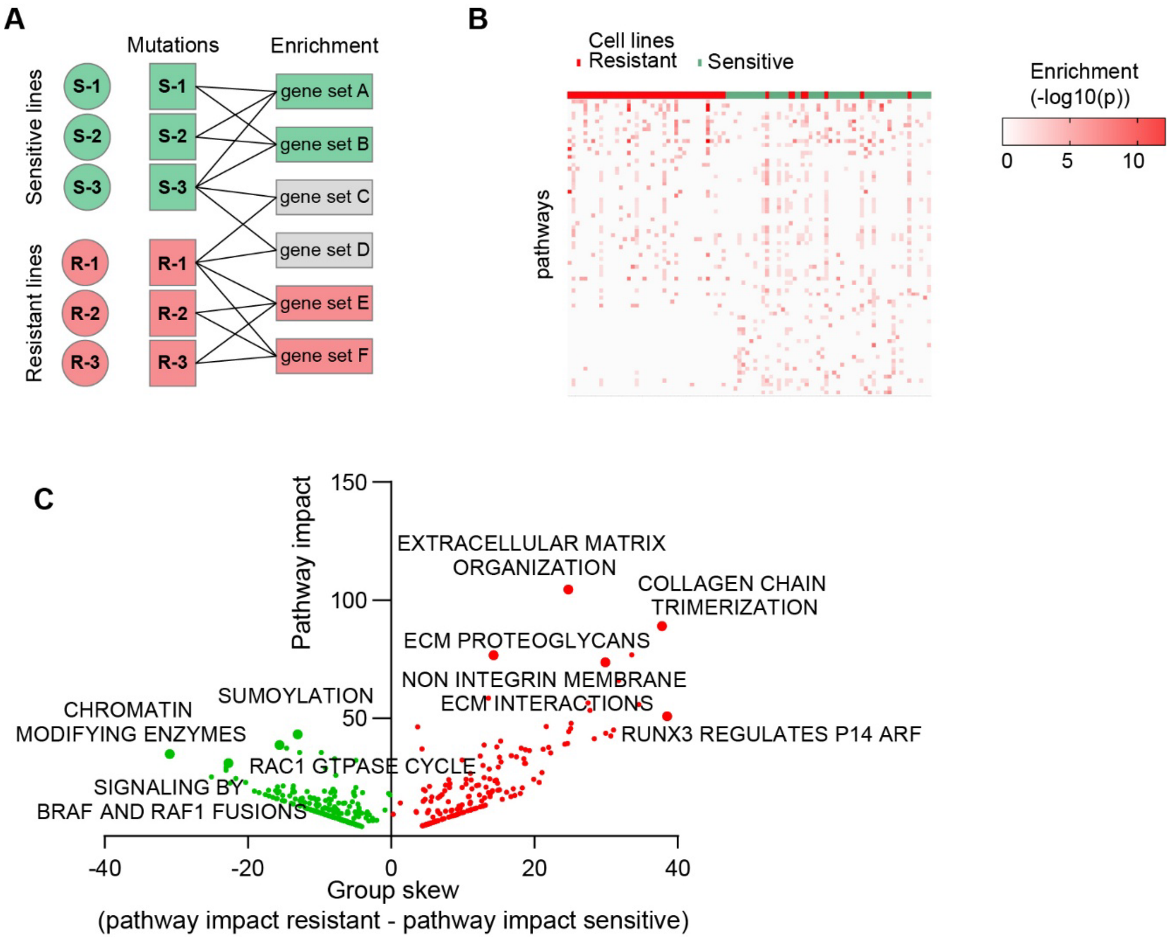

Supplemental Figure S3 (related to Figure 3). Alternative strategy for the identification of mutations associated with drug resistance

(A) Schematic of pathway-level mutation burden scoring per cell line.
(B) Heatmap of pathway enrichment scores across resistant and sensitive lines with hierarchical clustering (Ward's method, Euclidean distance).
(C) Volcano plot summarizing pathway-level group skew and significance, highlighting pathways enriched in sensitive or resistant lines. Group skew calculated as: (pathway impact in resistant lines) - (pathway impact in sensitive lines), where pathway impact is = (% of lines with enrichment) * (average enrichment score (-log10(P))).

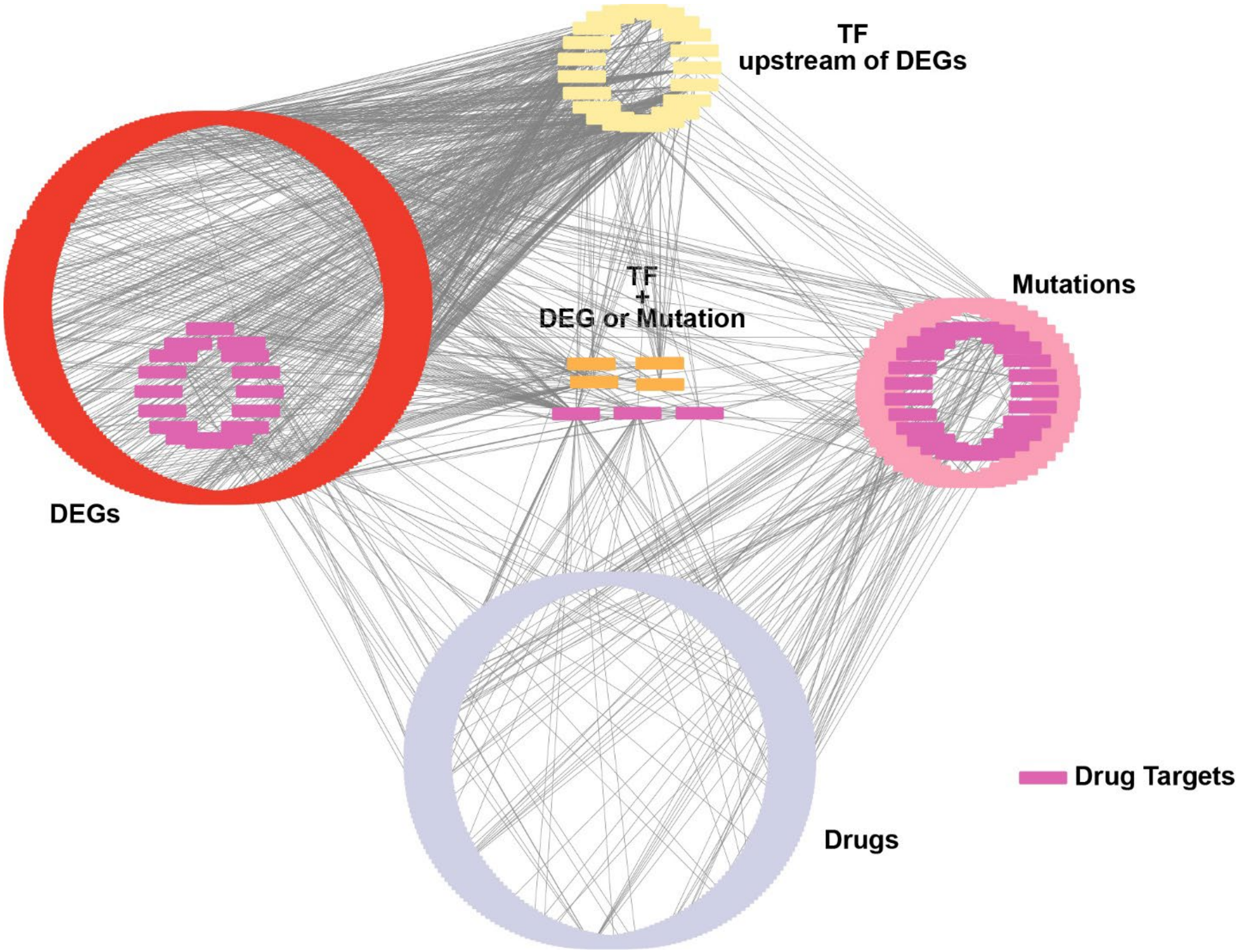

Supplemental Figure S4 (related to Figure 5). Full integrated drug-gene network

Integrated drug–gene network mapping candidate compounds to resistance-associated genes and transcription factors. Cytoscape interactions (edges) based on DGIdb drug-gene (nodes) interactions, and PPIs between genes. Full network, without labels.

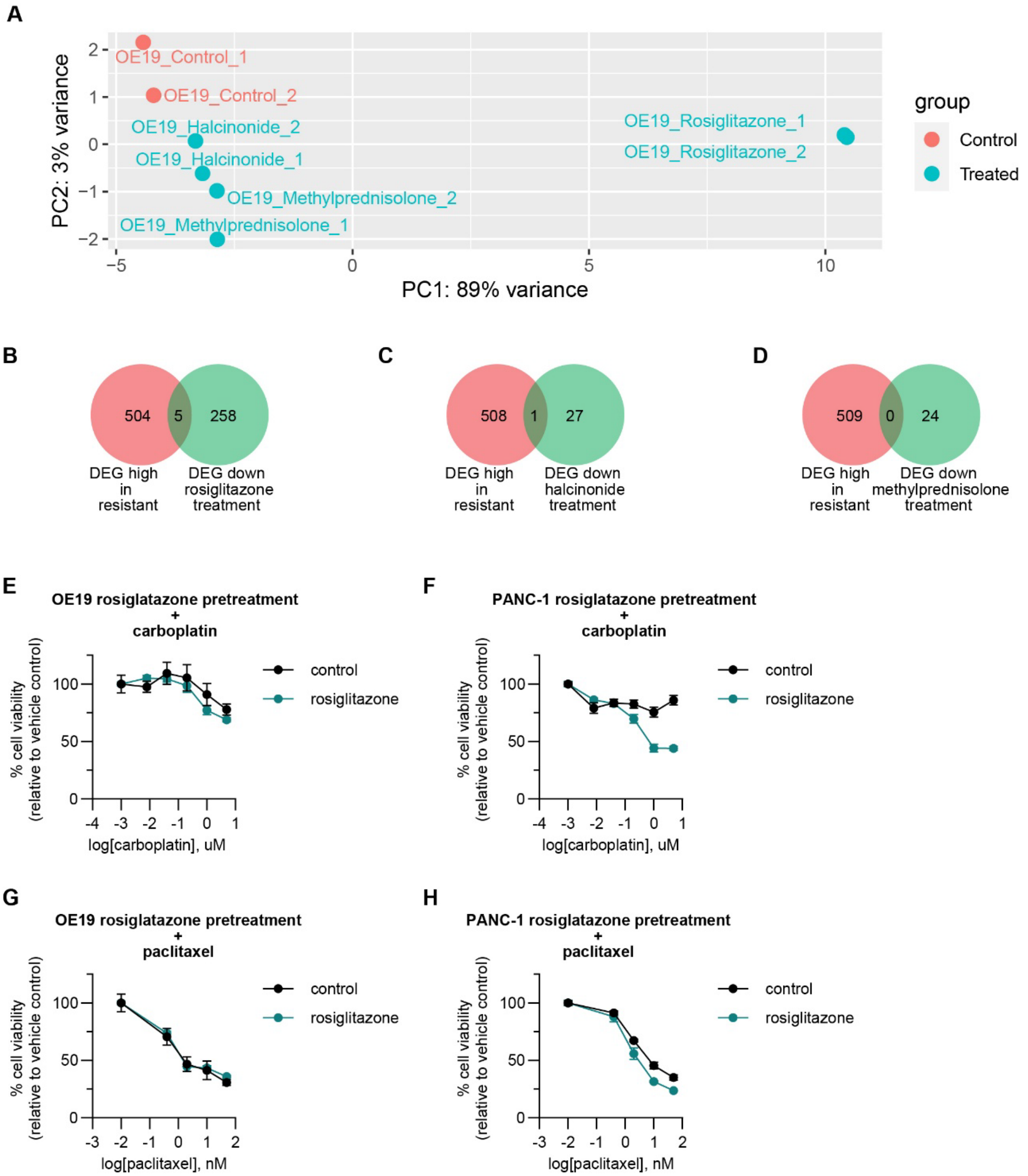

Supplemental Figure S5 (related to Figure 6). Additional experimental validation data to support predicted perturbagen screening

(A) Principal component analysis (PCA) of RNA-seq data from OE19 cells treated with halcinonide, methylprednisolone, or rosiglitazone (1 μM, 24 h; n=2 per condition).
(B–D) Venn diagrams showing overlap between genes upregulated in resistant lines and genes downregulated after perturbagen treatment using a stringent cutoff ($|\log_2 FC| > 1$, $P < 0.05$). Few overlapping genes were observed for halcinonide (n=1) and methylprednisolone (n=0), and a limited number for rosiglitazone (n=5).
(F) Functional enrichment analysis (Reactome) of DEGs downregulated by rosiglitazone. Overlapping or mechanistically related pathways to those enriched in resistant lines (see Fig. 2F) are highlighted, including vesicle trafficking, Rho GTPase signaling, ECM and platelet activation, stress-response (TP53/TGF-β) signaling, and nucleotide metabolism.
(E–H) Dose–response curves for carboplatin and paclitaxel in OE19 (E and G) or PANC-1 (F and H) cells pretreated (1 μM, 24 h) with rosiglitazone or vehicle, curves represent mean ± SD (n=4)
